# Structural Materials: Identification of the Constitutive Models and Assessment of the Material Response in Structural Elements Strengthened with Externally-Bonded Composite Material

**DOI:** 10.3390/ma13061272

**Published:** 2020-03-11

**Authors:** Todor Zhelyazov

**Affiliations:** 1 Structural Engineering and Composites Laboratory-SEL, Reykjavik University, Reykjavik IS-101, Iceland; elovar@yahoo.com; 2 Department of Mechanics, Technical University of Sofia, Sofia 1000, Bulgaria

**Keywords:** FRP, concrete, damage, synergy, strengthening, finite element analysis

## Abstract

This article investigates the material behavior within multiple-component systems. Specifically, a structural concrete element strengthened to flexure with externally-bonded fiber-reinforced polymer (FRP) material is considered. Enhancements of mechanical performances of the composite structural element resulting from synergies in the framework of the multiple-component system are studied. The research work comprises the determination of the constitutive relations for the materials considered separately as well as the investigation of materials’ response within a complex system such as the composite structural element. The definition of the material models involves a calibration of the model constants based on characterization tests. The constitutive relations are integrated into the finite element model to study the material behavior within the multiple-component system. Results obtained by finite element analysis are compared with experimental results from the literature. The finite element analysis provides valuable information about the evolution of some internal variables, such as mechanical damage accumulation. The material synergies find expression in the load-carrying capacity enhancement and the delay in the damage accumulation in concrete.

## 1. Introduction

This article investigates the structural response of materials considered either separately or as constituencies of a multiple-component composite system. The prediction of the behavior of a complex system is not always a straightforward task and requires the implementation of sophisticated techniques.

For the identification of multiple-component systems, one of the available approaches is the study of the mechanical wave propagation. The velocities of the compressional and shear waves depend on the elastic moduli of the continuum. Therefore, the estimation of these velocities is a basis of evaluation and testing of engineered materials (e.g., concrete) [1]. Specifically, the estimate of the ultrasound velocity provides information about material properties such as rigidity [2] stress state [3], and damage state [4].

For soft elastic materials, the study of wave propagation also has a number of practical applications. Among others, these are non-destructive defect detection [5,6] and acoustic tomography [7]. Some sources in the literature also report the investigation of inclusions-induced scattering of sound waves within the modeling of fiber-reinforced composites [8,9]. The mechanical properties of fiber-reinforced polymers (FRP) depend strongly on the inclusions, specifically on the volume fraction and spatial orientation of the fibers in the polymer matrix. The evaluation of the effective dynamic mass density of composites implies in-depth discussions [10,11,12,13]. Recently developed analytical frameworks [14,15], modeling the interaction and propagation of acoustic waves in soft, elastic material with scatterers (inclusions), allow for taking into account the form of the inclusions as well as their arrangement in the medium. Some literature sources report finite element analyses of the interaction between propagating sound waves and elastic structures embedded in the continuum [16,17]. Generally, the inclusions are either hard (steel, carbon) [18] or voids [19].

Given the complexity of the problem, reliable characterization procedures are necessary for the definition of the material models and the constitutive laws. 

The behavior of the multiple-component system can be characterized by a remarkable synergy of the constituent materials. This hypothesis should be verified for different cases. On the one hand, the material synergies enhance the performances of an FRP-strengthened reinforced concrete beam in terms of increased load-carrying capacity and stiffness. One the other hand, negative effects due to material interaction might be also present in some cases. A well-known example is the so-called premature failure of FRP-strengthened structural elements [20,21,22,23,24,25]. Global failure might result from a local failure in concrete and might occur even if the FRP reinforcement still has significant reserves in strength. In this case, the prediction of the material response based on a characterization test of the composite material considered separately appears to be not adequate within the multiple-component system.

Adding external FRP reinforcement (i.e., increasing the number of bonded FRP plates) leads to an increase in the load-carrying capacity. The flexural performance enhancement of concrete/reinforced concrete beams strengthened with externally bonded FRP plates pioneered by [26], has been investigated in numerous research works (see among others [21,22,27,28,29,30,31,32,33,34,35]). Recently, researchers also focus on the investigation of optimal design of FRP strengthened beams [36] and on new solutions, such as near-surface mounted FRP reinforcement [37]. Another novelty is the adding of discreet steel fibers in concrete: the materials’ synergies inhibit cracking and lead to further enhancement of the mechanical performances of the composite structural element [38]. In these techniques, material synergies enhance the mechanical performances of the composite structural elements. For a quasi-static loading, synergies result in a “delayed” global failure. They possibly modify the time rate of change of the damage accumulation. The verification of such a hypothesis requires quantification of degradation in concrete. The damage-based approach used in this study provides such an estimate.

The complex mechanical behavior of a composite structural element, specifically, a reinforced concrete beam strengthened to flexure with adhesively-bonded composite material, is the research object of this study. Assuming that the behavior of the composite structural element essentially depends on failure mechanisms in concrete, the investigation focuses on the strain-softening concrete response against a background defined by the other materials’ (FRP, adhesive, steel) responses.

Finite element simulation can provide valuable insight into the evolution of some internal variables (e.g., the accumulated damage), which are difficult to measure experimentally. Material models, needed as an input for the finite element analysis, are generally defined based on experimental data. Model constants are identified in numerical procedures designed to search for the set of constants providing the best fit with experimental data. The following materials characterization tests are required to build a numerical model of an FRP-strengthened structural element: (a) compression tests on concrete cylindrical specimens; (b) tension by flexure tests on concrete prismatic specimens; (c) tension tests on steel specimens (for the internal steel reinforcement); (d) tension tests on specimens made of the adhesive; (e) tension tests on specimens of the composite material plate. The identification of the response of the composite material/concrete interface requires additional tests, such as the single-lap shear test [39,40,41].

Complex systems may include “unknown” parameters [42]. In the context of the present study, the term system, or a “multiple-component system,” means a composite structural element made of components (i.e., materials) which exhibit various types of behavior but which complement each other to form the overall response of the structural element. From a certain point of view, the strengthened structural element can also be referred to as a “complex system.” For example, the premature failure modes that prevent the full utilization of the externally bonded FRP reinforcement are experimentally identified, typically, for specific geometry and external reinforcement arrangement. Also, the “delay” in the damage accumulation in concrete (within the multiple-component system), leading to an enhancement of the mechanical performances of the composite structural element, needs more in-depth analysis.

A numerical model is built to predict the complex behavior of the composite structural element. The model assumes experimentally identified constitutive laws to simulate constituent materials. The model is verified through a comparison of numerical results with experimental data. For an illustrative example, the experimentally-obtained data on the evolution of the mid-span deflection is compared with predictions on the evolution of the same parameter obtained by finite element analysis. In this context, the finite element analysis sheds light on the interaction between different components of the system (i.e., the different materials). A constitutive relation defined based on damage mechanics fundamentals is chosen to model the behavior of concrete and to investigate the synergy effect improving structural performance. This approach provides an insight into the evolution of some internal (or non-observable) variables, such as the damage accumulated in the representative volume element. On the basis of the distribution of this variable obtained (for example) by finite element analysis, an accurate assessment of the resources remaining in the material subjected to a specified loading history can be assessed.

## 2. Materials’ Behavior: Constitutive Relations

The behavior of materials is defined through the stress-strain relationship:

(1) $$\sigma_{i}=\sum_{j=1}^{6}C_{ij}\varepsilon_{j},\;i\;=1\ldots 6$$

In Equation (1), $\sigma_{i}$ are the stress components, $C_{ij}$ is the stiffness matrix, and $\varepsilon_{j}$ are the strain components. The stress-strain relationships, in their turn, are defined on the basis of empirical data obtained by identification tests on standard specimens. Therefore, to identify the response of an FRP-strengthened structural element, as a minimum, material characterization tests should be performed on specimens made of composite material, concrete, steel, and adhesive.

The formulation of the employed material models, as well as the identification tests used as a basis for their definition, are discussed in this section.

### 2.1. Composite Material

The FRP materials can be used as external reinforcement (sheets and plates externally bonded to the tensioned surfaces) or as internal reinforcement (reinforcing bars of filament in composite material analogous to the traditional steel reinforcement). 

**Figure 1:**
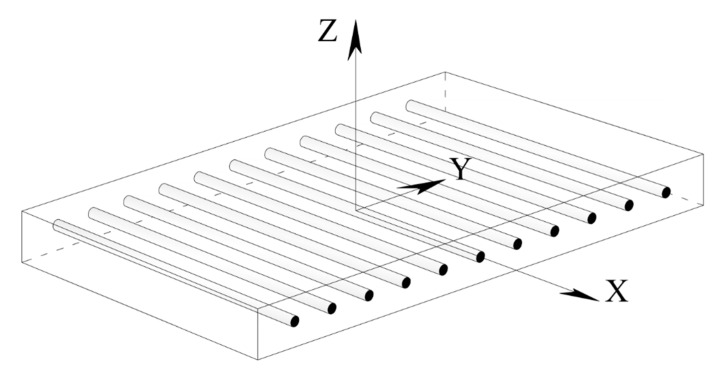
A model of unidirectional FRP plate.

It is known from experiments that the mechanical response of the FRP materials is linear and elastic until failure. However, there might be some nuances in the modeling of the FRP material’s behavior in the function of the material symmetries. For example, the unidirectional FRP sheets should be modeled by employing transverse isotropy. Thus, for a material in which the y–z plane is a plane of symmetry (x-, y- and z- are material directions: see Figure 1), the stiffness matrix is given by [43]:

(2) $$C_{ij}=\left(\begin{array}{cc} \begin{array}{ccc} C_{11} & C_{12} & C_{12}\\ C_{12} & C_{22} & S_{23}\\ C_{12} & C_{23} & C_{22} \end{array} & \begin{array}{ccc} 0 & 0 & 0\\ 0 & 0 & 0\\ 0 & 0 & 0 \end{array}\\ \begin{array}{ccc} 0 & 0 & 0\\ 0 & 0 & 0\\ 0 & 0 & 0 \end{array} & \begin{array}{ccc} \frac{C_{22}-C_{23}}{2} & 0 & 0\\ 0 & C_{55} & 0\\ 0 & 0 & C_{55} \end{array} \end{array}\right)$$

Taking into account that the stiffness matrix is the inverse of the compliance matrix:

(3) $$C_{ij}=S_{ij}^{-1}$$

the components of the stiffness matrix can be expressed in terms of the components of the compliance matrix as follows:

(4) $$C_{11}=\frac{S_{22}S_{33}-S_{23}^{2}}{S},C_{12}=\frac{S_{13}S_{23}-S_{12}S_{33}}{S},\;\;C_{22}=\frac{S_{33}S_{11}-S_{13}^{2}}{S},C_{23}=\frac{S_{12}S_{13}-S_{23}S_{11}}{S},\;C_{55}=\frac{1}{S_{55}}$$

where $S=S_{11}S_{22}S_{33}-S_{11}S_{23}^{2}-S_{22}S_{13}^{2}-S_{33}S_{12}^{2}+2S_{12}S_{23}S_{13}$. It should be noted that the elements of the compliance matrix are determined more directly than those of the stiffness matrix (see, for example, [44]).

**Figure 2:**
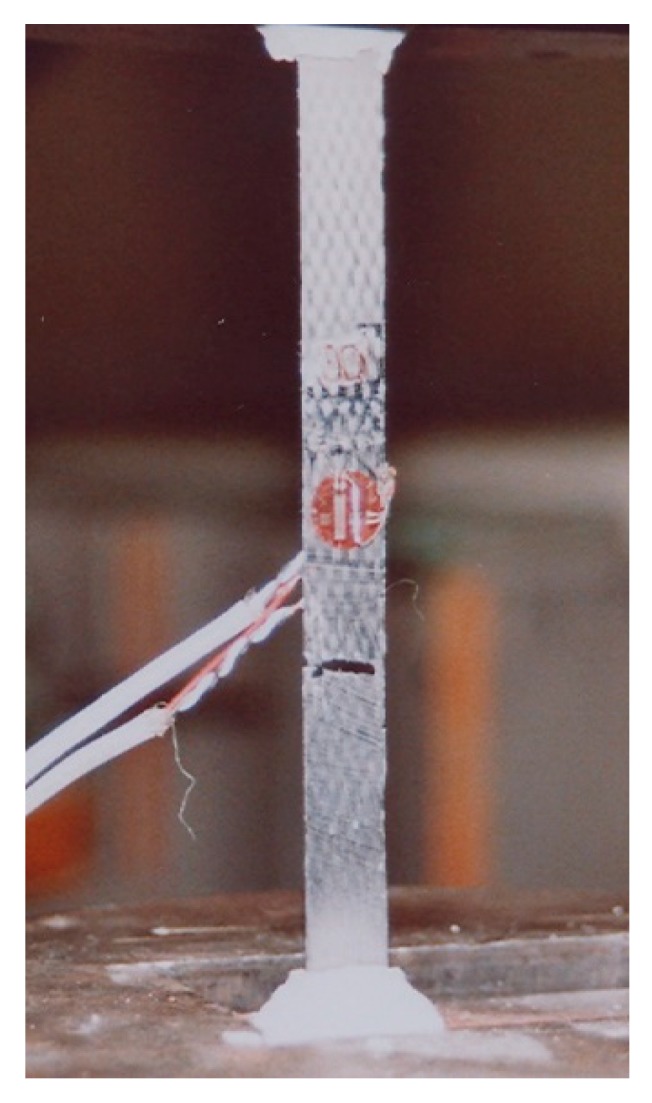
A test specimen of composite material: identification test [45].

The components of the compliance matrix are identified by performing tension tests on specimens made of composite material. Loading in different material directions is simulated by varying the angle between the applied load direction and the orientation of the fiber. Figure 2 shows a test specimen made of carbon fiber reinforced polymer (CFRP) and loaded in a direction parallel to the fiber orientation. The identified components of the elasticity tensor of the transversely isotropic profile are summarized in Table 1.

### 2.2. Concrete

Two approaches are possible to model the inelastic concrete response through the assessment of the accumulated damage. The elasticity tensor can be directly related to the current damage state, or the compliance matrix can be considered as a variable characterizing of the state of damage [46].

Within the first of the above-mentioned approaches, the inelastic mechanical response of concrete is modeled based on the stress-strain relationship of a linear, elastic material:

(5) $$\sigma_{i,j}=\frac{\nu }{\left(1+\nu \right)\left(1-2\upsilon \right)}E\varepsilon_{kk}\delta_{ij}+\frac{1}{\left(1+\upsilon \right)}E\varepsilon_{ij}$$

by adding a damage variable [47,48]:

(6) $$\sigma_{i,j}=\frac{\nu }{\left(1+\nu \right)\left(1-2\upsilon \right)}E\left(1-D\right)\varepsilon_{kk}\delta_{ij}+\frac{1}{\left(1+\upsilon \right)}E\left(1-D\right)\varepsilon_{ij}.$$

In Equations (5) and (6), *E* and ν denote, respectively, Yong’s modulus and the Poisson’s ratio respectively, $\varepsilon_{kk}$ is the trace of the strain tensor, and $\delta_{ij}$ is the unit tensor. The evolution of the damage variable in tension (*D_t_*) and compression (*D_c_*) is defined as follows [49]:

(7) $$D_{t}=1-\frac{\varepsilon_{inf}\left(1-A_{t}\right)}{\varepsilon_{eqv}}-\frac{A_{t}}{e^{B_{t}\left(\varepsilon_{eqv}-\varepsilon_{inf}\right)}}\;,$$

(8) $$D_{C}=1-\frac{\varepsilon_{inf}\left(1-A_{C}\right)}{\varepsilon_{eqv}}-\frac{A_{C}}{e^{B_{C}\left(\varepsilon_{eqv}-\varepsilon_{inf}\right)}}\;.$$

As can be seen, the evolution of either of the components of the damage variable is governed by the so-called equivalent strain:

(9) $$\varepsilon_{eqv}=\sqrt{\sum^{}\left\langle \varepsilon_{i}\right\rangle {}^{2}}.\;$$

The equivalent strain retains only the positive components of the principal strains $\varepsilon_{i}$ by employing the Macaulay brackets:

(10) $$\langle \varepsilon_{i}\rangle =\{\begin{array}{c} \begin{array}{ccc} 0 & if & \varepsilon_{i}<0 \end{array}\\ \begin{array}{ccc} \varepsilon_{i} & if & \varepsilon_{i}\geq 0 \end{array} \end{array}.\;$$

This feature of the model is in line with the phenomenological observation that cracking in concrete takes place in regions where tensile strains (stresses) are present. From a physical standpoint, the equivalent strain is related to the crack opening mode I [50]. In other words, cracking is generally related to tensile strains and stresses.

The behavior of concrete in compression was identified by testing standard cylindrical specimens (80 mm in radius and 320 mm in height) whereas the behavior of concrete in tension—by testing prismatic concrete specimens (400 mm in length and a cross-section of 100 × 100 mm; tension by flexure test).

**Figure 3:**
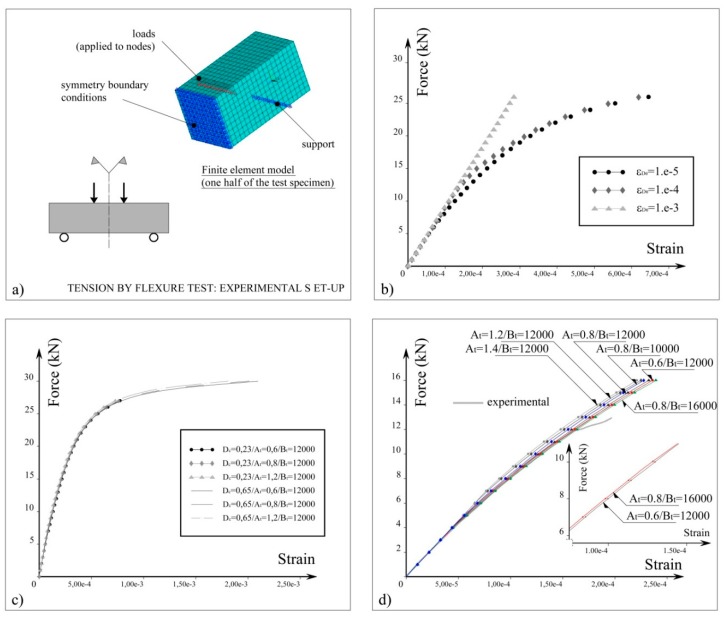
(**a**) Finite element model employed in the material characterization procedure and identification of the model constants; (**b**) dependency of the force-strain relationship on the damage threshold $\varepsilon_{D,0}$ (FE simulation of a compression test); for the simulation of the four-point bending tests, a value of the damage threshold is set to 5e-6.; (**c**) tuning of the material constants *A_c_* and *B_c_* (FE simulation of a compression test); (**d**) calibration of the constants *A_t_* and *B_t_* (tension by flexure test).

The identification tests are used to characterize the concrete response not only by determining compression strength, tensile strength, and maximum strain, but also to identify the model constants in Equations (7) and (8). These constants are identified through the curve fitting procedure. To this end, finite element models designed to simulate identification tests, specifically compression tests on cylindrical concrete specimens and tension by flexure tests on prismatic concrete specimens (Figure 3a), are built. The model constants are calibrated with respect to the experimental data. The constants $\varepsilon_{inf}$ (i.e., the damage threshold, see Figure 3b), *A_c_*, and *B_c_* are needed for the evaluation of the compression component of the damage variable (see Equation (8) and Figure 3c). The constants *A_t_* and *B_t_* are employed to define the evolution of the tensile component of the damage variable (Equation (7) and Figure 3d).

### 2.3. Steel

Steel response is characterized by an initial elastic phase as long as stress remains within the elastic domain. Upon reaching the yield stress, plastic flow is initiated. For common grades of steel reinforcement, it is followed by a hardening phase. A standard model based on the plasticity theory fundamentals is used [51,52]. In the large-displacements domain, a plastic behavior of steel reinforcement is expected. In this context, a true stress-strain curve is used, as a more representative measure of the state of the material, compared to the engineering stress-strain curve. The employed constitutive relation for steel uses an additive decomposition of the strain tensor into an elastic ($\varepsilon_{el}$) and plastic ($\varepsilon_{pl}$) part:

(11) $$\varepsilon =\varepsilon_{el}+\varepsilon_{pl}.$$

The strain tensor increment can be obtained as a sum of elastic and plastic increments:

(12) $$d\varepsilon =d\varepsilon_{el}+d\varepsilon_{pl}.$$

The yield criterion is a scalar function of the stress (σ) and a set of internal variables (ξ):

(13) f(σ,ξ )=0

The state variables at the mesoscale can be divided into observable (such as the total strain tensor) and internal (i.e., the elastic strain tensor, the plastic strain tensor, and the back strain tensor) variables [53].

Equation (13) defines the yield surface in the considered stress space. The evolution of the plastic strain is determined by the flow rule:

(14) $$d\varepsilon^{pl}=d\lambda \frac{\partial Q}{\partial \sigma }$$

where *Q* is the plastic potential, and dλ is the plastic strain increment. For kinematic hardening, the yield surface is defined by the following equation:

(15) f(σ−α,ξ)=0

**Figure 4:**
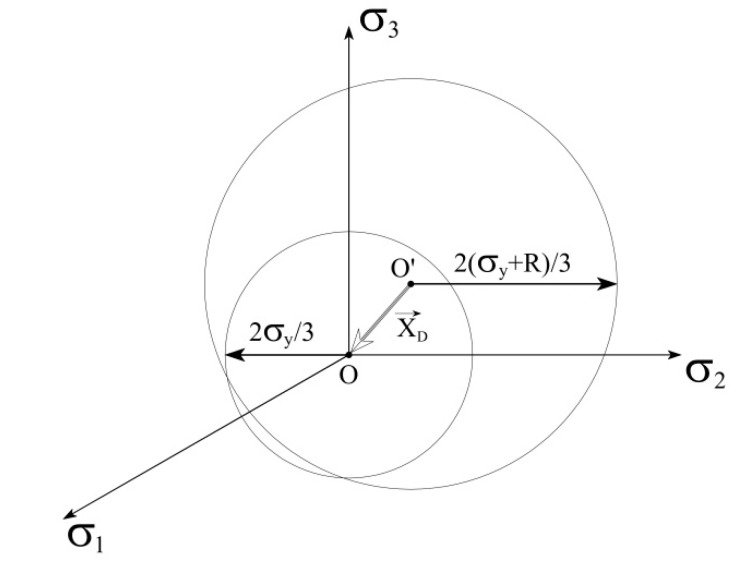
Geometrical interpretation of the back stress tensor.

In Equation (15), α denotes the back stress tensor (that can be interpreted as a vector *X_D_* pointing to the origin of the yield surface in the stress space; see Figure 4).

## 3. Behavior of Materials as Components of a System

The material constitutive relations, formulated based on characterization tests, are implemented into the numerical model of a multiple-component system. In the present study, this is a reinforced concrete beam strengthened to flexure by adhesively bonded composite material. The behavior of the multiple-component system is also studied experimentally. The model reproduces the geometry and the reinforcement arrangement (both internal steel reinforcement and external CFRP reinforcement) of the test specimen. It is validated based on a comparison between numerical and experimental results on the evolution of the mid-span deflection.

### 3.1. Finite Element Model

**Figure 5:**
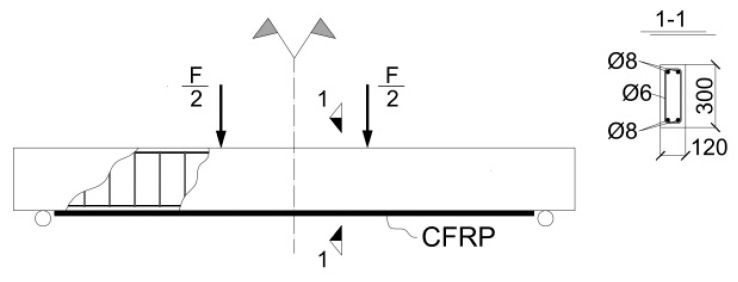
Geometry of the reinforced concrete beam strengthened with externally bonded CFRP and subjected to four-point bending test.

Figure 5 shows the geometry of the modeled (and of the experimentally tested) specimens. The employed material models are listed in Table 2. 

Taking into account the existing symmetries of the experimentally tested specimens, a quarter-model space is considered. Appropriate symmetry boundary conditions are applied to nodes located in the symmetry planes. In the finite element (FE) model, boundary conditions are applied to lines (an idealized situation). Nodes attached to the line of the (idealized) contact between the roller support and the beam, are constrained to move in the global y-direction. One of these nodes is also constrained in global x-direction. Forces in the global y-direction are applied to nodes attached to lines defined with respect to the application point of the concentrated load (the z-coordinates of these nodes is equal to the z-coordinate of the applied load). The sum of the magnitudes of these forces equals the current value of the applied load.

A perfect bond is assumed for all the interfaces (steel/concrete and concrete/CFRP).

In the 3-D FE model, the concrete, the steel bars, steel stirrups, and the adhesive layer, are meshed with Solid 95 (3-D 20-node structural solid). Shell 186 (3-D 20-node layered structural solid) is used to model the external CFRP reinforcement. The simulation of damage accumulation should take into account the effect of localization (localization of damage). A possible option is to remesh damaged regions with a finer mesh. With the implementation of higher-order elements containing mid-side nodes, the need for mesh refinement during analysis can be avoided. On the other hand, higher-order finite elements are a favorable option if the solution implies a refinement of the initial finite element mesh in specified regions. Additionally, the use of finite elements with mid-side nodes is a prerequisite for more accurate analysis results because of the improved shape function.

**Figure 6:**
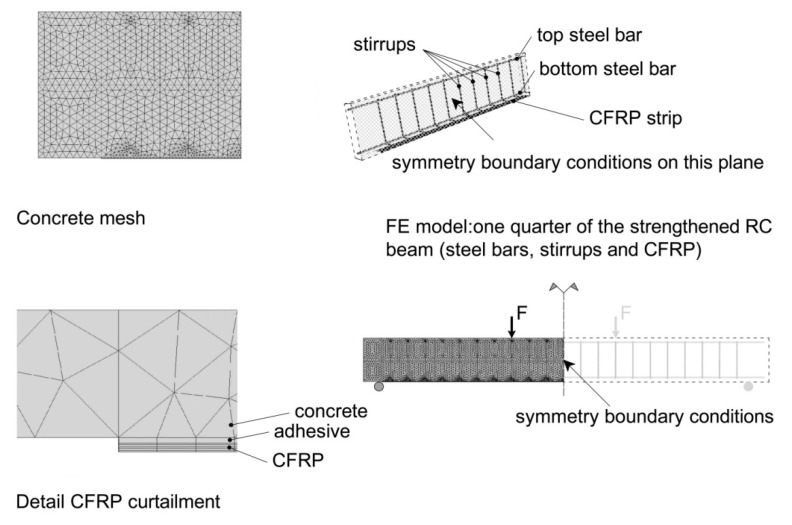
RC beam strengthened to flexure with externally bonded FRP material: finite element mesh.

With the hypothesis that the global response of the composite structural element is influenced mainly by the failure behavior of concrete, a finer mesh is generated for concrete. Additionally, the finite element size should comply with the requirements of the representative volume element size derived based on the energy balance in the framework of the continuum damage mechanics [53]. The sizes of the finite elements generated for CFRP reinforcement, the adhesive layer, and the steel reinforcement is defined so that a coherent mesh is obtained (i.e., smooth transitions between regions of different finite element sizes). Details on the generated finite element mesh, displayed in Figure 6, are summarized in Table 3.

The quasi-static loading is modeled by incrementally increasing the applied load. For each step of the applied load, a nonlinear static solution is performed. The damage-based constitutive relation for concrete is applied in the post-processing phase of each step. The damage variable is calculated based on the stress and strain distributions obtained in the finite element solution for the current value of the applied load. Before the next step of the solution, the material properties of the finite elements affected by damage are modified. Moreover, the finite elements in which the critical damage is reached are deactivated. In the next step, they don’t contribute to the overall rigidity. Thus, with the simulation of the propagating cracks, the finite element model becomes eventually insufficiently constrained. It is no more possible to perform nonlinear static analysis, and the numerical routine stops.

Finite element simulations reproduce force-controlled tests. From an experimental standpoint, displacement-controlled tests generally provide more accurate and more reliable results. However, the focus here is on the accuracy of the results obtained by finite element simulation, assessed through a comparison with the experimental data.

### 3.2. Prediction of the Transient Mechanical Response of the Composite Structural Element: Validation

**Figure 7:**
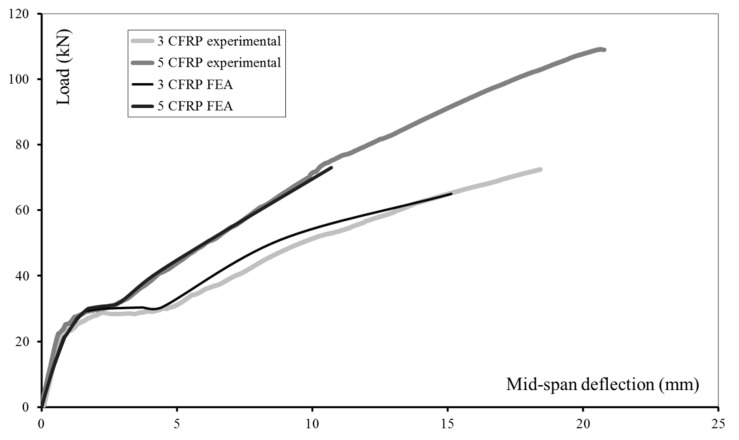
RC beam strengthened with three and five layers of externally bonded CFRP reinforcement: experimental results [45] and results obtained by finite element analysis (FEA).

Figure 7 shows experimental results and results obtained by finite element analysis. The numerical models reproduce the geometry, the reinforcement arrangement, and the material properties of experimentally-tested beams [45]. From the experimental data available, for finite element simulations are selected the beams strengthened with three and five layers of CFRP. As shown in Figure 7, such a difference in the amount of the externally bonded reinforcement illustrates well the effects of synergy in the composite structural element.

By assuming a specified value for the thickness of the FRP fabric (for example, *t_FRP_* = 0.2 mm), the cross-section of the FRP plate would be:

(16) $$A_{FRP}=n\times t_{FRP}\times b_{FRP}$$

where *n* is the number of FRP layers and *b_FRP_* stands for the thickness of the FRP plate. This cross-section can be further used in the design of the FRP-strengthened concrete element. In the present study, finite elements with the corresponding number of layers and material properties model the CFRP reinforcement.

The compliance between the numerical and experimental results is estimated as follows [54]:

(17) $$f=\left(1-\frac{\sqrt{\sum_{i=1}^{N}{\left(F_{n,i}-F_{e,i}\right)}^{2}}}{\sqrt{\sum_{i=1}^{N}{\left(F_{e,i}-F_{m}^{\left(e\right)}\right)}^{2}}}\right)\times 100$$

In Equation (17), “*F_n_*” and “*F_e_*” denote the vectors containing numerical and experimental results respectively, *N* is the number of components of the compared vectors, and $F_{m}^{\left(e\right)}$ is the mean of the components of the vector *F_e_*:

(18) $$F_{m}^{\left(n\right)}=\frac{\sum_{i=1}^{N}F_{e,i}}{N}.\;$$

A higher value of *f* corresponds to a better fit with the experimental data. The comparison between experimental and numerical results shows that for the beam strengthened with three layers of CFRP, the value of *f* is 75%, and for the beam, strengthened with five layers of CFRP—93%. 

The good agreement between experimental data and FE results (Figure 7) shows that the model can be employed in the analysis of problems involving the interaction of multiple material models, depending on a variety of factors. In a simplified analysis, for example, based on the effective moments of inertia of the composite element’s cross-section, three phases can be identified in the evolution of the mid-span deflection [55]. In the first phase, deflection develops until flexural cracks initiate in the tensioned zone of the cross-section. Al materials behave linearly within the elastic domain. The second phase (initiated with cracking in concrete) continues until the yielding stress in the internal flexural steel reinforcement is reached. The damage accumulation in concrete provokes nonlinearities in the global response of the composite structural element: the smooth transition between phase 1 and phase 2 depicted in Figure 7. Damage accumulation results in crack initiation and propagation in concrete. Cracks (upon intersection) activate the flexural steel reinforcement and the CFRP reinforcement. Moreover, in the cracked regions, in the tensile zone, only steel and CFRP contribute to the overall bending resistance. Between cracks, synergy provided by the concrete may be taken into account. With the progressive cracking in concrete and the failure of the tensile steel reinforcement in the cracked regions, only the CFRP reinforcement provides bending resistance in the tensile zone. The compression zone was not considered in the analysis of the other two phases. Taking into account the highly asymmetric (tension-compression) concrete response, at the beginning of phase three, concrete in the compression zone still has significant reserves in load-carrying capacity. The overall behavior of the structural element is governed by the composite material - linear and elastic until failure.

The analysis based on the effective moments of inertia is discussed here with the only purpose to provide an intuitive classification of the different phases in the response of a composite structural element subjected to a quasi-static loading. 

The analysis of both experimental and numerical results (Figure 7) leads to the conclusion that the performances of the FRP-strengthened structure element are enhanced in terms of load-carrying capacity (delayed damage accumulation with the increase of the amount of the external FRP reinforcement). The failure load increase demonstrates the enhancement of the load-carrying capacity. The delay in the damage accumulation is associated with the increase in the load corresponding to the end of the initial elastic phase. The synergy of materials can explain these observations.

As can be seen in Figure 7, the model of the multiple-component system provides accurate results in good agreement with experimental data for most of the loading history. The transitions between the initial elastic phase and the subsequent phase of increasing displacements, without a significant increase in the applied load, as well as the initiation of the hardening phase in the overall time history, are well captured. However, in the numerical solution, the load-carrying capacity of the CFRP-strengthened RC beam is underestimated. Indeed, *F_max,n_* = 0.9 × *F_max,exp_* where *F_max,n_* is the failure load predicted by the numerical simulation and *F_max,exp_* is the failure load obtained in the experiment. In the numerical analyses, the failure load and the maximum displacement are less than those obtained in the experimental study. This underestimation is referred to as a “premature numerical failure.” It can be attributed to excessive distortions in the initially generated finite element mesh. One possible solution to this problem could be the generation of new finite element mesh based on the current position of nodes, choosing one of the steps preceding the step at which the routine stops. The development of such an algorithm is the subject of ongoing research. Possibly, after testing, it will be possibly integrated into the analyses of structures’ responses within the large displacement domain. On the other hand, a modification of the solution options (number of steps and substeps) might increase the accuracy of the predictive results in the last phase of the loading history. A detailed and in-depth explanation of the premature, numerical failure also requires the extension of the database available. A conclusion made based on a large number of comparisons between numerical and experimental results would be more relevant.

**Figure 8:**
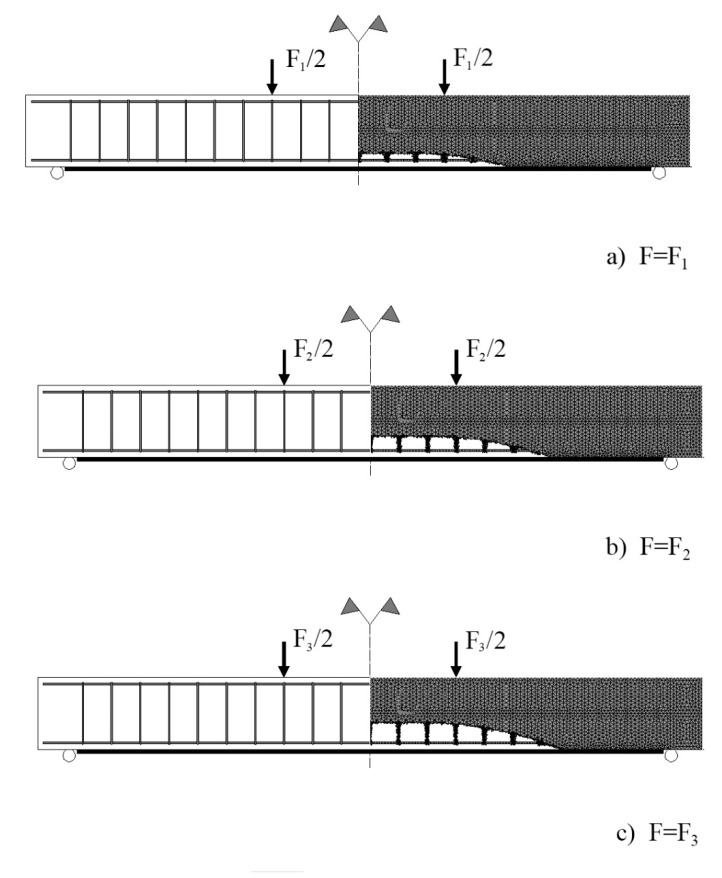
Finite element simulation of the damage propagation in concrete.

The strategy chosen to simulate the strain-softening concrete response, see Equations (6)–(8), involves the calculation of damage variable for each increment of the driving force parameter and for each finite element used to model concrete. Technically, the calculated damage variables are stored in predefined arrays. This algorithm makes possible the simulation of the initiation and propagation of the damage in an initially undamaged, homogeneous, and isotropic medium. In Figure 8, the propagation of the damaged zone in concrete is visualized. For three consecutive values of the applied load (*F*_1_ < *F*_2_ < *F*_3_), finite elements of a particular degree of mechanical damage are not displayed. Damage is initiated in the vicinity of the mid-span of the strengthened beam and propagates towards the supports.

With the increase of the applied load, mechanical damage accumulates, and the damaged zone propagates in concrete. When the damage variable, calculated for a given finite element, reaches a predefined critical value, the procedure deactivates this finite element in the next step of the incremental solution. As a result of the application of this algorithm, zones of zero rigidity nucleate and propagate in concrete. In the finite element simulation, the behavior of the flexural steel reinforcement and the externally bonded CFRP reinforcement intersecting a new-formed zone of zero rigidity (i.e., a crack) is analogous to their responses within an experimental study. Strains in CFRP and steel, within the regions of the intersection with cracks, are higher to resist the tensile force.

The finite element analysis sheds light on the debonding failure mechanism. Analysis of previous (mainly experimental) research leads to a classification of the debonding failure modes into two types [56]. Debonding of the external FRP reinforcement can be initiated at or near one of the plate ends and then propagates away from the plate end. Alternatively, debonding failure mode initiates at an intermediate flexural or flexural-shear crack and then propagates towards the plate end. Based on the visualization of the damaged zone propagation obtained by finite element analysis, it can be concluded that the failure mode triggered in the modeled beam is “intermediate crack induced interfacial debonding.” The interface failure depends on a variety of factors, such as the roughness of the surfaces in contact [57], exposure to aggressive environments [58], etc. With the hypothesis of a perfect interface (what excludes the above mentioned two failure modes), debonding results from a failure in concrete, in the vicinity of the adhesive joint.

## 4. Conclusions

The mechanical behavior of structural materials considered as elements of a multiple-component system has been investigated. Material models have been defined based on empirical data obtained in material characterization tests. 

A finite element model has been built to shed light on the unknowns related to the composite action: synergies in the materials’ response and material failure behavior within the structural element. The numerical model has been validated through a comparison with experimental data. The comparisons showed that the numerical model predicts with an adequate accuracy the experimentally-obtained evolution of the observable variable. The verification results demonstrate the capability of the numerical model to reproduce accurately the strain-softening concrete response taking into account the behavior of the other materials (FRP, adhesive, steel). The discussion focuses on concrete behavior since it is estimated that the global failure of the studied structural element results from a local failure in concrete.

A specific feature of the proposed approach is the possibility to track the evolution of the damage accumulation in concrete, which is directly related to the initiation and propagation of cracks on the macroscopic scale. Based on this monitoring, the load-carrying capacity and stiffness of the composite structural element remaining after a specified period of exploitation can be rationally estimated.

In the reported study, the enhancement of the mechanical performances of the composite structure demonstrates the synergy of the constituent materials. The external adhesively bonded CFRP reinforcement is a factor that enhances the load-carrying capacity of the strengthened beams. Also, it results in the delay in the failure mechanisms taking place in concrete.

## Figures and Tables

**Table 1:** CFRP mechanical properties [45].

E_x_	E_y_ = E_z_	υ_xy_ = υ_xz_	υ_yz_	G_xz_
(MPa)	(MPa)	-	-	(MPa)
120,000	27,800	0.35	0.18	4860

**Table 2:** Constitutive laws for constituent materials.

Material	Material Symmetries	Constitutive Law
Concrete	Isotropy	Elasticity coupled with damage
Steel	Isotropy	Bilinear kinematic hardening
CFRP	Transverse isotropy	Linear elasticity

**Table 3:** Size of the finite element mesh.

Component	Element Type	Element #	Node #
Concrete	SOLID 95	158,717	238,920
Steel	SOLID 95	4604	35,053
Adhesive layer	SOLID 95	1890	14,228
CFRP	SOLID 186	1890	14,868
